# Recent Updates in Pharmacological Properties of Chitooligosaccharides

**DOI:** 10.1155/2019/4568039

**Published:** 2019-10-31

**Authors:** Ilias Marmouzi, Shahira M. Ezzat, Maha M. Salama, Rana M. Merghany, Aisha M. Attar, Ahmed M. EL-Desoky, Shanaz O. Mohamed

**Affiliations:** ^1^University Mohammed V in Rabat, Faculté de Médicine et Pharmacie, Laboratoire de Pharmacologie et Toxicologie, Équipe de Pharmacocinétique, Rabat, Morocco; ^2^Pharmacognosy Department, Faculty of Pharmacy, Cairo University, Kasr El-Ainy Street, Cairo 11562, Egypt; ^3^Department of Pharmacognosy, Faculty of Pharmacy, October University for Modern Sciences and Arts (MSA), 6th October 12611, Egypt; ^4^Department of Pharmacognosy & Medicinal Plants, Faculty of Pharmacy, The British University in Egypt, 11837 Cairo, Egypt; ^5^Department of Pharmacognosy, National Research Centre, Giza, Egypt; ^6^Faculty of Science and Techniques, Hassan II University of Casablanca, Mohammedia, Morocco; ^7^Department of Molecular Biology, Genetic Engineering and Biotechnology Research Institute (GEBRI), University of Sadat City (USC), Sadat City, Egypt; ^8^Natural Wellness Scientific Office, Kuala Lumpur, Malaysia

## Abstract

Chemical structures derived from marine foods are highly diverse and pharmacologically promising. In particular, chitooligosaccharides (COS) present a safe pharmacokinetic profile and a great source of new bioactive polymers. This review describes the antioxidant, anti-inflammatory, and antidiabetic properties of COS from recent publications. Thus, COS constitute an effective agent against oxidative stress, cellular damage, and inflammatory pathogenesis. The mechanisms of action and targeted therapeutic pathways of COS are summarized and discussed. COS may act as antioxidants *via* their radical scavenging activity and by decreasing oxidative stress markers. The mechanism of COS antidiabetic effect is characterized by an acceleration of pancreatic islets proliferation, an increase in insulin secretion and sensitivity, a reduction of postprandial glucose, and an improvement of glucose uptake. COS upregulate the GLUT2 and inhibit digestive enzyme and glucose transporters. Furthermore, they resulted in reduction of gluconeogenesis and promotion of glucose conversion. On the other hand, the COS decrease inflammatory mediators, suppress the activation of NF-*κ*B, increase the phosphorylation of kinase, and stimulate the proliferation of lymphocytes. Overall, this review brings evidence from experimental data about protective effect of COS.

## 1. Introduction

Marine sources constitute nearly the half of the nature biodiversity and are considered the largest remaining reservoir of natural structures of food functional interest. Among marine food molecules, chitooligosaccharides (COS) are derivatives of chitosan with a lower molecular weight (MWt) [1]. The bioactivity and physicochemical properties of COS is highly dependent on their chemical characteristics. COS are produced by degradation of either chitin or chitosan *via* physical hydrolysis, acid hydrolysis, and enzymatic hydrolysis [2]. These oligomers usually present a degree of polymerization (DP) of <50–55 and a molecular weight (MWt) of <10,000 Da [3], and also they are characterized by a degree of deacetylation (DD), which is related to GlcN molar fraction in the compound, MWt distribution (polydispersity), and *N*-acetylation pattern (PA) [4]. In contrast with chitosan, the COS have higher solubility in water and their viscosity is low, so they are partially soluble in dimethyl sulfoxide and methanol but insoluble in ethanol and acetone [3]. COS have been traditionally obtained by chemical digestion with strong acids. However, many difficulties are associated with the traditional processes. In this regard, the use of chemical, enzymatic, or microbial hydrolysis is commonly employed for COS production. Degradation of chitosan using enzymatic hydrolysis has more benefits than using chemical hydrolysis regarding controllability and predictability [5–7]. Chitosan may be particularly degraded by chitinases, chitosanases, and lysozymes [3]. Even though chitinases and chitosanases obtained from microbes produce a relatively higher proportion of COS, their usage is limited due to their elevated price. So, many nonspecific enzymes are used, including cellulases, pectinases, lipases, amylases, and deacetylases [2, 8]. Moreover, COS having certain PA pattern may be generated by bacterial deacetylases [9]. COS generated by these enzymes are mainly consisted of a combination of oligomers with different PA, DD, and DP. Recently, COS have received much attention in biomedical and food industries mainly because of their solubility, biocompatibility, biodegradability, and nontoxicity. COS had broad range of applications and proved diverse promising biological activities, and subsequently, they drew attention in drug therapy [6,10]. This review focuses on marine COS as a therapeutic strategy against diabetes, oxidative stress, and inflammation. The pharmacokinetic profile and the molecular and biochemical regulatory pathways of these molecules are discussed with updated bibliographic data.

## 2. Search Methodology

Electronic searches were performed in PubMed, Scopus, Google Scholar, and Web of Science databases, using the following keywords: chitooligosaccharides, chitosan-oligomers, and chitooligomers, to identify relevant articles. All published articles were evaluated, and a total of 103 papers of important contribution were selected to compose the present review.

## 3. Results and Discussion

### 3.1. COS Antioxidant Properties

COS exhibit antioxidant properties *in vitro* and *in vivo*; the literature data reporting antioxidant COS are summarized in Tables 1 and 2. COS exhibited strong scavenging activity on the HO^•^ and O_2_^•^ radicals [11]; however, their activity is relatively weak on alkyl and DPPH radicals. In this work, the activity was related to the concentration, MWt, and tested radicals. Haung et al. [12] modified COS into two derivatives by introducing carboxyl and quaternary amino groups to their amino positions with varying substitution levels. The aim of this modification was to change the ability of hydrogen atoms' whole quantity to react with free radicals and make them more capable to chelate ion metals. COS were also previously prepared from different molecular weights using incomplete chitosan hydrolysis, and their free radical scavenging activity was estimated and correlated to their MWt and compared to aminoglucose [13]. The antioxidant action of COS, vitamin C, and aminoglucose was dose-dependent in a phenazine methosulfate-*β*-nicotinamide adenine dinucleotide system, though it was nonlinear. Yang et al. [13] suggested that COS amino groups may have an efficient task in their antioxidant action due to their short chains, regarding chitosan which forms well-built intramolecular and intermolecular bonds and reduces the reaction of the free radicals with the amino and hydroxyl groups of the polymers. Further investigation of COS with different MWt (<1000 Da) on intracellular radical (H₂O₂, ^•^OH, and ROO^•^) using B16F1 and murine melanoma cell line [14] demonstrated marked decrease in those radical species supporting the concept that a lower MWt is essential for the radical scavenging activity in biological systems. Also, COS low MWt suppressed NF-*κ*B gene promoter activity, proving their ability to avoid diseases associated with oxidative stress. Lee et al. [15] evaluated the protective effects of sulfated chitosan and COS by employing a fluorescence-activated cell sorter (FACS) on hydrogen peroxide and plasmid DNA strand breaks assay. The sulfated chitosan and sulfated COS (S-COS), having highest alkyl radical scavenging activity, were selected to investigate their protective liver cell effect against H_2_O_2_. Results proved that the S-COS III showed the highest protective activities on liver cells and exhibited a protective effect on H_2_O_2_-induced DNA damage. Fernandes et al. [16] studied the free radical scavenging action of two COS combinations and a low MWt LM-chitosan against two biological molecules, phages and erythrocytes, that underwent different oxidative stress situations. The potential inhibition of COS for each tested concentration was higher than that of LM-chitosan. Higher concentrations of COS potentiated the H_2_O_2_ action; in contrast, LM-chitosan potentiated H_2_O_2_ effect, at lower concentrations. In addition to the previous mechanisms, either COS or LM-chitosan had the capability to keep RBCs from oxidation of Hb by AAPH at conc. of 0.05–0.005 mg/mL. Fernandes et al. [16] concluded that COS may be utilized as antioxidants *in vivo*, at concentrations moderately low to stop more oxidative injury to cells, though this was not in agreement with chemical methodology. Ngo et al. [17] studied the effect of gallic acid conjugated to COS (gallate-COS) on cellular antioxidant activity. The mechanism of the antioxidant activity of gallate-COS was assessed by measuring the levels of gene expression of GSH and SOD antioxidant enzymes. Analysis of the results showed that gallate-COS reduced free radical-catalyzed activation of NF-*κ*B protein (this is a transcription factor-mediated expression of several genes in osteoarthritis condition). Accordingly, gallate-COS may be utilized as a potent antioxidant to protect bone cells from oxidative stress. Lu et al. [18] investigated the antioxidative role of S-COS opposing H_2_O_2_-mediated injury in pancreatic MIN6 *β*-cell line. Results revealed that S-COS were safe at conc. of 0.1 to 0.5 mg/mL. Moreover, S-COS can augment the viability of cells, decreasing ROS generation and decreasing the LDH and MDA in oxidative damaged *β*-cells demonstrating its antioxidant effect. The suggested mechanisms are due to the S-COS improvement of activity of antioxidant enzymes, reduction of ROS generation, and inhibition of MIN6 *β*-cell apoptosis. Likewise, increasing the DS has an impact on the defense mechanisms against oxidative damage. Many studies estimated the different antioxidant action of LM-polysaccharides (LMPS) prepared by starch (LMST), agar (LMAG), and chitosan (LMCH), on fibroblasts of skin and correlated the activity regarding their amine, hydroxyl, and sulphur groups. The studies reported that the variation in antioxidant activity of LMPS might be because of LMPS functional groups, ability to chelate metal ions, ability to transfer electrons, and capacity to stabilize radicals. This high capacity to stabilize radicals and metal ion chelating property of LMCH resulted in a potent antioxidant action that is advanced to that of LMAG. So, LMCH showed a high ability to reduce ROS generation, reduced lipid peroxidation of the cells, and inhibited DNA damage due to oxidative stress. Accordingly, they reported that LMCH may have an efficient role in treating skin complications and can be used as sun protective agents [19]. Other reports indicated that low MW COS exhibited the highest antioxidant activities [20].

Recently, Qu and Han [39] investigated COS antioxidant action *in vivo* using high-fat diet (HFD) mice model. Mice groups were administered normal diet, HFD, or HFD + COS (0.5%) for a period of 6 weeks. Group fed with HFD +0.5% COS significantly restored the action of GPx, CAT, and SOD in liver, stomach, and blood when comparing it with the HFD-administered group. Morphologically, there was a significant decrease in the measured parameters: decreased villus height compared with the group fed with HFD. However, those fed with HFD + COS (0.5%) were the same in the height of villus compared with the control. This result supports the improvement in intestinal integrity when COS was added to their diet. Furthermore, results showed that gallate-COS had a defensive potential regarding H_2_O_2_-mediated DNA oxidative harm [21]. Gallate-COS reduced the generation of reactive oxygen species inside H_2_O_2_-mediated A549 cells. On the other hand, Xie et al. [40] proved that supplementary maternal diet with COS enhanced blood SOD level, which resulted in decreasing MDA content. Also, expression of some antioxidative genes mRNA was enhanced in the mothers' placenta. Jiang et al. [41] also reported the *N*-acetyl chitooligosaccharides can attenuate amyloid *β*-induced damage by reducing the oxidative stress in animal and cell models of Alzheimer's disease.

### 3.2. COS Anti-Inflammatory Effect

COS are promising anti-inflammatory agents (Table 3). LM-COS comprising of glucosamine (GlcN) *n*, where *n* = 3–5, were fit for restraining both antigen-invigorated degranulation and cytokine production in rodent basophilic leukemia cells (RBL-2H3) [53]. Moreover, the defensive impact of LM-COS regarding ovalbumin-incited lung irritation in asthma mouse model (16 mg/kg/day) was also established [53]. Dung et al. [54] demonstrated that COS upregulated the declarations of CD86 and MHCII on SDCs and advanced the emission of TNF-*α*. Additionally, nonsoluble COS invigorated the differentiation of the CD4+T in dendritic cells. COS were examined for their consequences for epithelial cells as well as tissues [55]. Chitooligomers fundamentally invigorated the mitochondrial action of *in vitro* keratinocytes' cultures. Supplementation with COS significantly reduces the intestinal provocative reaction, which is corresponding with the enactment of CaSR and the suppression of NF-*κ*B signaling pathway under inflammatory conditions [54].

COS produce an anti-inflammatory impact *via* Nrf2/MAPK-stimulated HO-1 production [56]. Kim et al. [57] showed that LM-S-COS suppressed the generation of nitric oxide and some inflammatory mediators like TNF-*α* as well as IL-6 in LPS-induced RAW 264.7 cells. Kunanusornchai et al. [58] indicated that COS may have *in vitro* as well as *in vivo* synovial anti-inflammatory activity through AMPK phosphorylation. Moreover, COS showed suppressor action on LPS-stimulated reduction in the Bcl-2/Bax fraction and higher activity of caspase-3 and BKCa [42]. One of the techniques that COS can perform to suppress apoptosis of cells is by controlling BKCa channel. Then again, COS can hinder the activation of LPS-prompted p38 as well as quicken the activation of O-GlcNAc glycosyltransferase enzyme. Li et al. [59] found that five chitooligomers from dimer to hexamer (chitobiose, chitotriose, chitotetraose, chitopentaose, and chitohexaose) separated from COS enhanced NF-*κ*B-related luciferase gene expression as well as inhibited NF-*κ*B gene transcription. COS reduced OGT-related NF-*κ*B O-GlcNAcylation and so inhibited LPS-mediated inflammation of the vascular endothelia [60]. Polar chitosan formulation based on nanoparticles CMC-COS NP and SC-COS NP was produced by the development of complexes of polyelectrolyte [61]. Injection of CMC-COS NP and SC-COS NP changed Th cytokine content in blood and induced the differentiation of lymphocytes in spleen *in vivo*, approving their ability to control cell-induced immune reactions. COS efficiently suppressed TNF-*α*-mediated activation of ICAM-1 and VCAM-1 at transcription as well as translation levels [62]. Liu et al. [62] found that COS suppressed LPS-mediated IL-8 activation in HUVECs *via* blocking p38 MAPK as well as PI3K/Akt pathways. COS decreased the generation of proinflammatory mediators such as IL-1*β* and nitric oxide in LPS-mediated RAW 264.7 cells [63]. COS may also defend inflammation and oxidative stress in LPS-mediated complications *in vivo*, which may present valuable properties for septic patients [63]. Results showed that COS decreased the contents of nitric oxide and PGE2 generation by inhibiting COX-2 and iNOS activation without considerable cytotoxicity in BV-2 microglia [64]. Notably, COS exerted anti-inflammatory effect through blockade of I*κ*B-a decomposition, NF-*κ*B translocation, and MAPK phosphorylation (dose dependent). Xu et al. [65] demonstrated that COS suppressed EGF-mediated cell migration *via* blocking EGFR/MAPK pathway. COS suppressed the proliferation of epithelia cells of the breast *via* deactivation of GnT-V as well as its products [66]. Nutritional supplements containing COS or GMOS increased IL-1*β* gene activation of mucosa in jejuna as well as lymph nodes, when compared to lincomycin supplements [67]. Yoon et al. [68] showed that COS can act as an anti-inflammatory agent through stimulating TNF-*α* in LPS-mediated inflammatory complications of RAW 264.7 cells. COS may also be efficient in treating inflammatory bowel disease *via* inhibiting NF-*κ*B pathway as well as apoptosis of cells of epithelia in intestine [69]. Zhang et al. [70] found that COS may possess potential stimulating incidence in the immune system by enhancing the production of TLR4 on macrophage while Zhang et al. [71] found that they efficiently suppresses the IL-1*β*-mediated chondrocytes' apoptosis through activation of p38 MAPK pathway. Dai et al. reported protective effect of COS against neuroinflammation *via* its inhibitory effect on BACE1 [22]. Moreover, COS have been shown to exhibit immunostimulatory effect *via* the MAPK and PI3K/Akt signaling pathways [72]. Another study demonstrated that fully deacetylated and acetylated COS exhibit an ability to reduce the level of TNF-*α* in murine macrophages after LPS stimulation [73].

### 3.3. COS Antidiabetic Mechanisms

COS exert its antidiabetic activity through different mechanisms (Table 4). In a study made by Liu et al. [82], the highly deacetylated COS (90%) of molecular weight 1200 Da obtained by chitosan enzymatic degradation using chitosanase enzyme. COS incidence on pancreatic islet cell viability as well as *β-*cell line using MTT colorimetric test was established. The COS sufficient entrance into cells of pancreatic islet was approved by COS-induced insulin secretion assays through utilizing cultures of rat monolayer islet cells, where COS at a concentration (100 mg/L) enhanced the differentiation of islets and induced recovery of damaged pancreatic *β*-cells increasing insulin release to 14 days, when comparing the results to the group of normal control. COS also promoted *β*-cell proliferation in pancreas (INS-1) and upregulated GLUT2 mRNA gene expression, which could stimulate insulin release. COS also can defend INS-1 cell against apoptosis [83] which occurs as a result of increasing the circulating glucose, saturated FA [84, 85], inflammatory markers [86], and oxidative stress [87–89].

Streptozotocin (STZ) is a molecule that may act as selective cytotoxic agent for pancreatic cells as well as generating ROS that results in DNA degradation [90, 91]. COS showed a protective effect on INS-1 cell in opposition to apoptosis mediated by STZ, through raising the activity of reduced SOD and reduction of elevated levels of malondialdehyde (MDA) content in pancreas homogenate [82, 83, 92]. On the other hand, COS may not be able to defend INS-1 cell against harm mediated by some proinflammatory cytokines, such as IFN-*γ*, TNF-*α*, or IL-1*β* that can enhance *β*-cell apoptosis *via* the intrinsic mitochondrial apoptotic pathway [93].

*In vivo* experiments in STZ-induced diabetes mellitus (DM) in mice showed that COS could decrease 2 h plasma glucose in 60 days at a dose of 500 mg/kg, to 16.14 mmol/L, when comparing the results to the diseased DM group at *P* < 0.01. This study also proved that the dose 500 mg/kg of COS also showed the best incidence in the oral glucose tolerance test (OGTT) through improving the sensitivity to insulin. Area under the curve (AUC) for the DM group was regarded as 100%, and AUC of normal control group was only calculated as 17.69% of the DM group AUC, so treatment with 500 mg/kg of chitooligosaccharides caused a decrease to 68.69% of the DM group AUC [82]. STZ-mediated type 2 DM results in insulin resistance as well as *β*-cell disordering [94]. In a study designed by Ju et al. [83], STZ-diabetic rats showed metabolic disorders including hyperglycemia, hyperlipidemia, insulin resistance, and ruptured islet. COS showed *in vivo* hypoglycemic effect at a dose of 1000 mg/kg, and it exerts its effect by reducing fasting serum glucose as well as insulin levels, increasing index of insulin sensitivity and improving OGT, protecting pancreatic islet, and reducing insulin resistance. COS also caused a considerable increase in glycogen level in liver, and this might be through increasing the transfer of blood glucose into liver glycogen by increasing glucokinase.

Chronic hyperglycemia may be acquired as a result of the failure of the body peripheral tissues to utilize glucose properly. GLUT-4 is a vital glucose transporter and regulator of its metabolism found in skeletal muscles and adipocytes [95, 96]. In this study, the expression of GLUT-4 was decreased in adipose and skeletal tissues in diabetic animal model. Treatment of the diabetic animals with COS caused a significant increase in GLUT-4; this effect may be attributed to COS which can upregulate GLUT-4 mRNA expression, thus improving insulin resistance. *In vivo* administration of COS in STZ-diabetic rats could also repair the injury of pancreatic tissues and restore the BWt ratio of pancreas. Liu et al. [82] suggested that COS being an alkaline help in decreasing the plasma glucose through increasing the pH of the body fluids and thus raise insulin sensitization. COS may also maintain normal metabolism of plasma glucose by regulating the endocrine system as well as reducing insulin secretion to standard levels. Administration of COS to alloxan-induced type II diabetes mellitus in mice at two doses 5 and 10 mg/kg decreased plasma glucose levels by 54.1% on day 21st of the experiment, significantly increased hepatic glycogen content, and gradually increased the body weight of the treated mice, while the diabetic mice continued to lose weight [97]. SGOT and SGPT activities were increased significantly in alloxan-induced diabetic mice [97]. This increased activity of transaminases occurs in the absence of insulin due to the increase of amino acids levels in the blood causing increased gluconeogenesis and ketogenesis [97]. SGOT and SGPT which act as indicators of liver function were come towards control level after treatment with COS (10 mg/kg); this indicates the normal liver function [97] COS (10 mg/kg) have also caused alteration in lipid metabolism and this was evidenced by decreasing the levels of serum LDL, VLDL, triglycerides, and total cholesterol levels which were elevated due to diabetes and increased HDL-c level [97]. Interestingly, these results from Ha et al. [98] study suggest that deep-sea water having COS showed a higher inducible result on glucose uptake more than either alone. Also, this consequence is accomplished by activating different signaling pathways associated with GSV trafficking. Yu et al. [99] showed that COS may have dual mechanism of actions as antidiabetic in intestinal cell lines, either through suppressing *α*-glucosidase and SGLT1 as well as GLUT2, or through improving adipocyte proliferation and activation of PPARγ as well as its targeted genes, like FABP4 and adiponectin, where these properties were enhanced through cotreating with BADGE (PPARγ antagonist). Also, COS considerably enhanced glucose uptake. *In vivo* COS administration did not demonstrate any antidiabetic/hypocholesterolemic effects, since glycemia and cholesterol levels in GK rats were not altered [100]. In a recent study [101], the consumption of COS with complexes to resistant starch produced a higher capability for insulin sensitivity revival. Gene expression of liver tissues obtained from COS-injected rats approved that this effect may be due to increase in the liver glucose alteration through activation of GS2 as well as GYG1, decrease in gluconeogenesis through activation of G6PC1, and change in glucolipid metabolism through activation of Insig-2. Also GlcNAc 2 decreased lipid peroxidation as well as inflammatory markers in pancreatic cells with enhancement of SOD activity and decreased IL-1b, NF-*κ*B, MDA, and TNF-*α* contents [102]. GlcNAc 2 may also appreciably deactivate MAPK pathway particularly through IL-1b-Erk/p38-histone H3 pathway in type 2 DM *in vivo*.

## 4. Current Evidence and Perspectives

Marine compounds with interesting *in vivo* antioxidant properties are excellent drug candidates against inflammation, diabetes, and oxidative stress. COS especially show interesting properties in experimental data. COS are generated from chitin deacetylation and hydrolysis reactions, through enzymatic or chemical methods. The COS are normally absorbed *via* the epithelia of the intestine and mainly dispersed to the hepatocytes, splenocytes, and renal cells. COS are also decomposed by lysozymes before being excreted *via* urine. Due to their high polarity, low viscosity, safety, and good pharmacokinetic profile, COS have already been integrated in many functional formulations, such as COS capsules. It is noteworthy that, among the several publications that focuses on COS bioactivity, many recent works detailed the molecular mechanisms and targeted signaling pathways of COS action. However, many mechanisms are not evidenced, mainly because of poor reproducibility, divergence in physicochemical properties, and dose effect. Among the proposed mechanisms are, e.g., the pancreatic islet proliferation, the increase in insulin secretion and sensitivity, the reduced postprandial glucose and improved glucose uptake, the upregulatory GLUT2 expression and inhibition of digestive enzymes and glucose transporters, the reduction of gluconeogenesis and promotion of glucose conversion, the decrease in inflammatory mediators and enzymes, and the suppression of NF-*κ*B pathway. Moreover, COS stimulate the proliferation of lymphocytes and radical scavenging activity and decrease ROS and reduce MDA in oxidative damaged cells. COS have been shown to increase intracellular antioxidant enzymes, prevent oxidative DNA damage, and inhibit or suppress superoxide anion and NO production. Interestingly, the bioactivity of COS is highly dependent on its physicochemical properties, which may result in new findings and bioactive compounds according to extraction and purification method and/or molecular weights. Therefore, optimization and controllable methods of extraction and polymer synthesis will contribute to reproduce relevant therapeutic effects. For example, decrease in COS MWt is related to their high antioxidative effects. Similarly, increases in DD and positive charges are related to their high anti-inflammatory as well as antioxidative activities. Chemical modifications of COS present a chance for enhancing different biological activities of COS. Future research should be directed towards the understanding of mechanism of action of standardized and chemically characterized COS. All together experimental data and processing technology provide an evidence of defensive potential of COS against glucose-mediated oxidative complications through being *in vivo* antioxidant, anti-inflammatory, and antidiabetic agents; COS can also regulate many signaling pathways involved in glucose metabolism and homeostasis. The safe profile of COS, diverse mechanism, and MWt-dependent bioactivity lead us to expect a wide range of applications for COS technological formulations.

## Figures and Tables

**Table 1:** *In vitro* antioxidant activities of COS.

Sample	Assay	Effect	Reference
COS	Hydroxyl and superoxide; alkyl and DPPH assays	Antioxidant activity of COS is related to their MWt and the free radical examined.	[11]

Modified COS with carboxyl and quaternary amino groups to the amino position	DPPH; inhibition of lipid peroxidation; Fe^2+^ chelating; carbon-centered radical scavenging assays	The radical scavenging activity is dependent on reactive hydrogen atom level, molecular charge property, and their capability to chelate metal ions.	[12]

COS Ch1100 and Ch500	Scavenging of superoxide ion by means of phenazine methosulfate (PMS)	Ch1100 is an effective free radical scavenger.	[13]

LM-COS	Intracellular radical scavenging effects	However, the antioxidant activity is dependent on MWt. COS efficiently protected the DNA not considering the MWt.	[14]

S-COS	Fluorescence-activated cell sorter (FACS) on H_2_O_2_ and plasmid DNA strand breaks assay	S-COS possesses powerful shielding effects on hepatic cells and DNA opposing H_2_O_2_-induced oxidation.	[15]

COS mixtures and LM-COS	ABTS assay; two natural oxidisable molecules, phages and erythrocytes, that underwent oxidative damage by H_2_O_2_	Decreased both the DNA and hemolytic damage, by reducing H_2_O_2_ and AAPH radicals, but not in a dose-dependent manner.	[16]

Gallyl-COS	Testing the antioxidant activity *via* an electron spin resonance method; DNA, protein, and lipid peroxidation in chondrosarcoma human cells	Reduce oxidative stress caused by free radicals.	[17]

S-COS with diverse substitutions	H_2_O_2_-mediated damage in pancreatic MIN6 *β*-cell line	Antioxidant activity of S-COS through reducing free radical generation and decreasing the content of MDA and LDH level in intracellular cells.	[18]

Gallate-COS	Cell viability; ROS production; DNA oxidation; DPPH assays using ELISA and western blot analysis	Efficient element with powerful anti-inflammatory and antioxidant activities.	[21]

COS	Estimation of free radicals *via* calculating lipid peroxidation level and caspase-3 activity	Keep the hippocampal neurons from A*β*-mediated neurotoxicity.	[22]

Acetylated COS	Measurement of intracellular ROS and the mitochondrial membrane potential	May act as antagonists opposing glutamate-mediated PC12 cell fatality.	[23]

Chitooligomers with several degrees of polymerization	Hydroxyl and superoxide radical scavenging assay; reducing power assay	Increase in superoxide ion scavenging action when degrees of polymerization increased.	[24]

S-COS	Estimation of nitric oxide generation and nitric oxide synthase action	Antioxidative effect opposing H_2_O_2_-mediated apoptosis in *β*-cells; prevention of mitochondrial pathway and transcription factor NF-*κ*B activation.	[18]

COS	MTT assay; determination of cell apoptosis using flow cytometry; measurement of ROS production; estimation of reduced GSH; transfection of small interfering RNA	Inhibition of ethanol-mediated oxidation of cells through enhancement of Nrf_2_ and decrease of phosphorylation of MAPK.	[25]

COS	Synthesis and characterization of paclitaxel-loaded COS-stabilized gold nanoparticles (PTX-COS AuNPs) and COS gold nanoparticles (AuNPs); free radical scavenging assay; viability of cells assay and photoacoustic tomography	Efficient action of PTX-COS AuNPs in drug delivery systems and acting as anticancers.	[26]

COS	Cell culturing and viability estimation; myeloperoxidase activity; protein oxidation assays; estimation of free radical-induced DNA oxidation by fluorescence probe 20, 70-dichlorofluorescein diacetate (DCFH-DA); measurement of intracellular GSH level	Inhibition of oxidative stress in live cells.	[27]

COS	Cell culturing and viability estimation; protein oxidation assays; estimation of free radical-induced DNA oxidation by determining GSH content	Promising antioxidant agent *in vivo*.	[28]

Gallate-COS	Estimation of lipid, protein, and DNA damage; cell free radicals by RNA separation and RT-PCR analysis	Reduction of oxidative damage of lipids, proteins, and DNA; reduce NF-*κ*B expression and enhance the content of antioxidant enzymes inside cells.	[29]

Chitooligomers	AChE activity; carboxylesterase activity; free radical scavenging activity estimation using DCFH; reduced GSH determination; dopamine content; investigation of deterioration in neurons of *C. elegans* transgenic strains; protein assessment by Lowry's method	Inhibition of the deterioration of dopaminergic neurons and related physiological changes mediated by monocrotophos *in C. elegans* through inhibition of cell oxidation.	[30]

Chitin-oligomers	Anticancer; oxidative DNA; intracellular free radical estimation; RT-PCR and western blot analysis	Prospective molecules to prevent neurodegenerative disorders.	[31]

*N*-acetylated COS	Hydroxyl radical scavenging assay; H_2_O_2_ scavenging assay; DPPH assay; assessment of protection of DNA damage	Preventing oxidative DNA damage in peripheral blood mononuclear cells exposed to H_2_O_2_.	[32]

COS coating on iron oxide nanoparticles	Cell culture and MTT assay for anticancer activity; acridine orange/ethidium bromide double staining assay; mitochondria damage assessment using transmission EM; detection of mitochondria membrane integrity; measurement of ROS by DCFH-DA and DHE	Reduction in oxidative cell harm and fair free radical generation.	[33]

*N*-carboxymethyl COS	DPPH assay; superoxide anion scavenging activity assay; reducing power assay	Antioxidant activities in antioxidant systems; inhibiting efficacy on superoxide anion.	[34]

COS	Culture of cells; drug treatment; viability of cells; intracellular NO measurements by confocal microscopy and flow cytometry; nitrate assay; western blot analysis and RT-PCR	Suppression of the generation of NO in LPS-mediated N9 murine microglial cells *in vitro*.	[35]

COS	Estimation of APAP and APAP conjugates inside plasma and hepatocytes; DME action; antioxidant enzymes; MRP2/3; western blot analysis	Reduction of acetaminophen-mediated hepatotoxicity through inhibiting CYP-induced bioactivation.	[36]

Acetylated COS	DPPH assay; reducing power; superoxide anion radical scavenging assay; hemolysis of erythrocyte assay; non-enzymatic protein glycation; assay of NBT reduction; assay of AGE measurement; determination of NO generation	Inhibitory effects on oxidation and glycation	[37]

4-Hydroxybenzyl-chitooligomers	Anticancer; DNA oxidative damage of Chang hepato cells; free radical estimation inside cells by FACS and light microscope analyses; RT-PCR, western blot analysis, and nuclear protein extraction	Prevention of H_2_O_2_-mediated oxidative damage of Chang hepato cells; enhancement of antioxidant enzyme content; suppression of ROS production, DNA oxidative damage, and NF-*κ*B signaling pathway.	[38]

**Table 2:** *In vivo* antioxidant activities of COS.

Sample	Assay	Effect	Reference
COS	High-fat diet (HFD) mouse model; superoxide radical scavenging activity; hydroxyl radical scavenging and DPPH assay	Reduction of the oxidative damage resulted from the HFD.	[39]

Chitooligomers with degrees of polymerization	Measurement of reducing power; hydroxyl radical scavenging assay	Increase of SOR scavenging potential of chitooligomers accompanying several degrees of polymerization.	[42]

COS	Estimation of O_2_ and H_2_O_2_; flow cytometric analysis to assess neutrophil apoptosis	Neutrophils' proapoptotic capacity from glycogen-mediated peritonitis in mice model; inhibition of SOR generation; reduction of the myeloperoxidase liberation.	[43]

COS	Histological study and retinal cell count; estimation of NeuN-positive ganglion cell layer neurons; terminal deoxynucleotidyl transferase-induced dUTP biotinide end labeling (TUNEL); cell culturing of RGC-5 and *in vitro* oxidative damage	Preventing ischemia of retina through decreasing oxidative and inflammatory conditions.	[44]

COS	Determination of GSH, MDA contents, and SOD activity; evaluation of neuronal apoptosis through TUNEL assay; immunohistochemical analysis of 8-OHdG, IL-1b, and TNF-*α*	Amyloid-1–42-induced rat model of Alzheimer's disease; antioxidant activity in hippocampus, effects on apoptosis (TUNEL assay); inhibition of neuroinflammatory responses.	[45]

Chitin, chitosan, COS and *N*-acetyl-D-glucosamine	Measurement of antioxidant defense parameters; determination of antioxidant enzyme activity; MDA and carbonyl protein assays	Dietary intake containing COS could enhance the growth performance of *P. monodon* and improve its resistance to DO stress on shrimp, *Penaeus monodon*.	[46]

COS	Measurement of antibacterial activities of COS; measurement for the resistance to COS after long-term culture; biochemical analysis; RT-PCR and western blot analysis	Reduction of antibiotics dose; prevention of antibiotics-caused side effects in adolescent idiopathic scoliosis (AIS) patients with spinal fusion surgery.	[47]

COS	Measurements of antioxidant-related indices; analyses of cytokines and immunoglobulins; duodenal, jejunal, and ileal histomorphological studies; intestinal mucosal digestive enzymes assay and sIgA; extraction of total RNA and reverse transcription reactions; PCR and microbial population determination	Enhancing growth performance, antioxidant capacity, immunity, and intestinal development of weaned pigs.	[48]

*N*-acetyl COS	DPPH assay and production of *N*-acetyl COS; measurement of enzyme activity	Production of antioxidants and *N*-acetyl COS by Serratia sp.; TKU020 fermentation.	[49]

COS	Drug-metabolizing enzyme activity; estimation of lipid peroxide and GSH levels and GSH S-transferase and NQO1 activity; western blot analysis	Suppression of hepatic CYP enzymes and enhancement of phase II detoxifying reactions of hepato and renal cells *in vivo*.	[50]

COS	Cell culture; nitrite assay; measurement of TNF-*α*; spectrofluorometric assay of RDPase; protein and creatinine assay; SOD and MDA assay	COS stimulated the production of TNF-*α*, NO, and RDPase.	[51]

COS	Measurement of antioxidant enzyme's activities or oxidant injury product; RNA isolation and real-time polymerase chain reaction	Increase of antioxidant defense capacity and placental amino acid transport of sows.	[52]

**Table 3:** Anti-inflammatory potential of COS.

Sample	Model	Reported activity	References
COS	*In vivo* paw edema rat model	The anti-inflammatory activity is related to COS dose and their MWt.	[16]

COS	BV-2 microglia	Inhibitory effects on generation of interleukin IL-1b, IL-6, and TNF-*α*; blocking degradation of I*κ*B-a inhibitor; transfer of NF-*κ*B and MAPK.	[21]

COS	Human umbilical vein endothelial cells	Inhibition of LPS-induced cell apoptosis; increase of caspase-3 and regulation of the conductance calcium-stimulated potassium channel.	[42]

COS	L9 microglial cells (*in vitro*)	Suppression of nitric oxide generation; inhibition of p38 MAPK phosphorylation and decreased AP-1 and NF-*κ*B activation.	[35]

COS	Autoimmune anterior uveitis model (*in vitro*)	Clinical score reduction; reducing the inflammatory markers such as MCP-1, iNOS, RANTES, and TNF-*α*.	[44]

COS	*In vivo* acute renal failure model	Antioxidative activity enhanced kidney tasks.	[51]

LM-COS	RBL-2H3 cells (*in vitro*) and ovalbumin-sensitized/challenged mouse asthma model (*in vivo*)	Decrease the generation and activation of inflammatory cytokines.	[53]

Soluble (S) and insoluble (B) COS	Spleen CD11c^+^ dendritic cells (SDCs)	B-COS induce SDC maturity, TNF secretion, and promotion of CD4+T proliferation; COS bioactivity depends on MWt or degree of polymerization.	[54]

*N*-acetyl-D-glucosamine oligosaccharides	*In vitro* and *ex vivo* skin epithelial cells and tissues; *ex vivo* GIT epithelial membranes	Activation of skin cells' differentiation; increasing the mucin secretion from GIT cells.	[55]

COS-supplemented diet	LPS-challenged piglets	Decrease the inflammation of the intestine, through CaSR activation and suppression of NF-*κ*B pathway.	[74]

COS	RAW 264.7 cells (LPS-activated murine macrophage)	Induction of HO-1 activation; reduction of iNOS and COX-2; activation of ERK1/2, JNK, and p38 MAPK signaling pathways.	[56]

S-COS (crab shells)	RAW 264.7 cells (murine macrophage)	Suppression of proinflammatory markers such as iNOS and NO.	[57]

COS (MWt∼5000 Da, DD ∼90%)	Rabbit and human synoviocytes	Induction of AMPK activation; increase in the ADP/ATP ratio; suppression of TNF-*α*-mediated COX-2 and iNOS activation through AMPK pathway.	[58]

Chitobiose, chitotriose, chitotetraose, chitopentaose, and chitohexaose	Cell line, 293T	Activation of NF-*κ*B-dependent luciferase genes and downstream of transcription of NF-*κ*B genes.	[59]

COS	Endothelial cells (cultured) and mice model	Suppression of LPS-induced NF-*κ*B-dependent inflammatory gene expression; decrease in OGT-dependent O-GlcNAcylation of NF-*κ*B; attenuation of LPS-stimulated inflammation.	[60]

COS nanoparticles	Mouse fibroblasts (3T6), HeLa cells, and melanoma cells (B16)	Induction of the proliferation of fibroblasts; modulation of Th cytokines; stimulation of spleen's lymphocyte proliferation.	[61]

COS	Human umbilical vein endothelial cells	Inhibition of TNF-*α*-stimulated activation of ICAM-1 and VCAM-1 at the translation and transcription stages; block the TNF-*α*-stimulated expression of NF-*κ*B; block the decomposition of I*κ*B-a and the activation of ERK1/2 and p38 MAPK; reduce the adhesion of U937 monocyte to HUVECs; suppression of ICAM-1 and VCAM-1 generation in activated HUVECs.	[62]

COS	Septic mice	Reduction of blood IL-1*β* and TNF-*α*; attenuation of p38-activated protein kinase and c-Jun NH_2_-terminal kinase.	[63]

COS	Epithelial GE11 cells	Epidermal growth factor (EGF)-induced epithelial GE11 cells growth inhibition; block EGFR phosphorylation and MAPK activation.	[65]

COS	Human breast epithelial cells (MCF-10A)	Inhibition of cell migration induction; suppression of GnT-V protein expression.	[66]

Galacto-mannan-oligosaccharides	Early weaned piglets	Enhance IL-1*β* gene activation in mucosa of jejuna and lymph nodes; improve the blood levels of IgM, IgG, IgA, IL-6, IL-2, and IL-1*β*.	[67]

COS	LPS-induced (RAW 264.7 cells)	Inhibition of LPS binding to TLR4/MD-2 receptor complex; attenuation of the stimulation of MAPK; decrease in NF-kB nuclear transmission; reduction in proinflammatory generation (IL-1, NO).	[68]

COS	ICR male mice; T84 cells (human epithelial cells of colon)	Suppression of stimulation of NF-*κ*B and contents of IL-6 and TNF-*α* in colon cells; failure of the epithelial barrier to function.	[69]

COS	RAW 264.7 macrophages. ICR mice	Enhance the phagocytosis by macrophages; increase the generation of nitric oxide and TNF-*α* by macrophages; increase the TLR4 and inducible iNOS mRNA levels.	[70]

COS	Sprague Dawley neonatal rats	Inhibit cell apoptosis; improve mitochondrial membrane potential and IL-1*β*-induced nuclear chromatin damage in chondrocytes; activate the p38 MAPK signaling pathway.	[71]

COS	Obese model (*in vivo*)	Reduction in the weight increase through inhibition of inflammation.	[75]

COS	Sepsis model (*in vivo*)	Decreased organ malfunction and enhanced the rate of surviving.	[76]

COS	BV-2 microglial cells (*in vitro*)	Decreased PGE2 and NO generation through suppressing the activation of COX-2 and iNOS; reduced the IL-1*β*, IL-6, and TNF-*α* contents; inhibited p38 MAPK and JNK activation.	[77]

COS	Human umbilical vein endothelial cells	Suppression of LPS-mediated IL-8 activation through blocking the p38 and Akt protein kinases.	[78]

COS	*S. aureus* isolated from mastitic cows	Antibacterial activity against *S. aureus*. Immunostimulatory effect; enhancement of nonspecific immunity cells through raising monocytes.	[79]

COS	Hybrid tilapia (*Oreochromis niloticus* × *Oreochromis aureus*)	Decrease the mRNAs encoding and TNF content; increase of transforming growth factor-b levels; reduction of *A. hydrophila* infection.	[80]

Deacetylated COS	RAW macrophages	Increase of the cell viability; moderate anti-inflammatory activity.	[73]

COS	IPEC-J2 (porcine intestinal epithelial cells)	Attenuation in activation of mRNA of MCP-1 and IL-8 stimulated through TNF-*α*; decrease of mRNA expression of claudin-1.	[81]

**Table 4:** Possible mechanisms by which COS exert their related antidiabetic actions.

Model	Antidiabetic mechanism	References
Suckling piglets	Upregulation of cholesterol accumulation in suckling by the regulation of circadian clock genes	[52]

Mice	Reduction of body weight raising and adiposity; improvement of abnormal blood and liver lipid profiles	[75]

MTT colorimetric assay on pancreatic *β*-cells (100 mg/L); STZ-induced diabetic rats treated with COS at 500 mg/kg	Accelerate differentiation of islet cells of the pancreas; increase insulin secretion from pancreatic *β*-cells; reduce postprandial glucose	[82]

Enzyme-linked immunosorbent assay on pancreatic *β*-cells (INS-1 cells) at 100 and 500 mg/L; STZ type 2 diabetic rat models, fed on high energy diet treated by means of COS at 1000 mg/kg	Protects INS-1 cells from STZ-induced apoptosis; upregulated GLUT2 mRNA gene expression; increased proliferation of INS-1 cells; improving insulin sensitivity index (ISI)	[83]

C2C12 myotubes	Improve glucose uptake in C_2_C_12_ myotubes, even in the absence of insulin	[98]

Intestinal cell line (Caco-2) and adipocyte cell line (3T3-L1)	Suppression of intestinal glucose transporters SGLT1 and GLUT2 and *α*-glucosidase enzyme; enhancing adipocyte differentiation through activation of PPARc and its target genes; increase of glucose uptake; reduce hyperglycemia through suppressing the absorption of glucose and its transport	[99]

Wistar and Goto–Kakizaki (GK) rats	No antidiabetic/hypocholesterolemic effects if glycemia and cholesterol levels in GK rats are not altered	[100]

High-fat diet fed rats	Reduction of gluconeogenesis through increasing the expression of G6PC1 gene; enhancement of glucose conversion in liver through increasing the expression of GYG1 and GS2 genes	[101]

Type 2 diabetic mice	Reduction of glucose and total cholesterol levels in blood through improving their metabolism; reversal of tissue resistance to insulin	[102]

3T3-L1 cells	Adipogenesis suppression	[103]

Adipocytes (3T3-L1)	Inhibition of adipocyte proliferation stimulated *via* inhibiting adipogenic transcription factors' expression	[104]

3T3-L1 adipocyte	Suppression of adipocyte proliferation *via* activation of PPARg and C/EBPa	[105]

Obese rats	Improve dyslipidemia and prevent body weight gains by inhibiting the differentiation of adipocyte	[106]

SD rat model	Suppression of *α*-glucosidase enzyme activity; enhance the absorption of glucose to cells of fat and muscle	[107]

Sprague Dawley rats	Reduce cardiovascular risk factor and atherogenic index through reducing TG, LDL, and T-CHO contents in blood and enhancing their excretion in feces	[108]

Alloxan-induced mice	Decrease of glucose level in blood by increasing the insulin secretion; decrease of SGPT and SGOT levels in blood; decrease of both cholesterol and triglyceride levels	[109]

Broilers	Decrease of LDL cholesterol without any alteration in HDL cholesterol	[110]

db/db mice model	Reduction of the blood glucose level and HbA1c; suppression of sucrose, maltase, and glucoamylase enzymes	[111]

Streptozotocin-induced diabetic rats	Reduction of blood glucose concentrations; decrease of glycated hemoglobin; increase of the plasma C-peptide and insulin secretion	[112]

ob/ob mice	Ameliorated levels of adipokines by activation of PPARg gene expression; increase in adiponectin level; decrease in resistin, IL-6, and TNF-*α* levels	[113]

Adipose tissue of ob/ob mice	Antiobesity effect; downregulation of gene expression of PPARg and SREBP-1c	[114]

High-fat diet fed rats	Reduced VLDL/LDL ratio and TG; increased the activity of LCAT in plasma	[115]

Suckling piglets	Reduced glucose level through enhancing gluconeogenesis in the liver	[116]
